# Autophagy and Apoptosis Induced in U87 MG Glioblastoma Cells by Hypericin-Mediated Photodynamic Therapy Can Be Photobiomodulated with 808 nm Light

**DOI:** 10.3390/biomedicines9111703

**Published:** 2021-11-17

**Authors:** Viktoria Pevna, Georges Wagnières, Veronika Huntosova

**Affiliations:** 1 Department of Biophysics, Institute of Physics, Faculty of Science, P.J. Safarik University in Kosice, Jesenna 5, 041 54 Kosice, Slovakia; viktoria.pevna@student.upjs.sk; 2 Laboratory for Functional and Metabolic Imaging, Institute of Physics, Swiss Federal Institute of Technology in Lausanne (EPFL), Station 6, Building CH, 1015 Lausanne, Switzerland; georges.wagnieres@epfl.ch; 3 Center for Interdisciplinary Biosciences, Technology and Innovation Park, P.J. Safarik University in Kosice, Jesenna 5, 041 54 Kosice, Slovakia

**Keywords:** glioblastoma cells, autophagy, photodynamic therapy, photobiomodulation, apoptosis, microscopy, hypericin

## Abstract

Glioblastoma is one of the most aggressive types of tumors. Although few treatment options are currently available, new modalities are needed to improve prognosis. In this context, photodynamic therapy (PDT) is a promising adjuvant treatment modality. In the present work, hypericin-mediated PDT (hypericin-PDT, 2 J/cm^2^) of U87 MG cells is combined with (2 min, 15 mW/cm^2^ at 808 nm) photobiomodulation (PBM). We observed that PBM stimulates autophagy, which, in combination with PDT, increases the treatment efficacy and leads to apoptosis. Confocal fluorescence microscopy, cytotoxicity assays and Western blot were used to monitor apoptotic and autophagic processes in these cells. Destabilization of lysosomes, mitochondria and the Golgi apparatus led to an increase in lactate dehydrogenase activity, oxidative stress levels, LC3-II, and caspase-3, as well as a decrease of the PKCα and STAT3 protein levels in response to hypericin-PDT subcellular concentration in U87 MG cells. Our results indicate that therapeutic hypericin concentrations can be reduced when PDT is combined with PBM. This will likely allow to reduce the damage induced in surrounding healthy tissues when PBM-hypericin-PDT is used for in vivo tumor treatments.

## 1. Introduction

Glioblastomas are among the most aggressive primary tumors of the central nervous system. They are characterized by aggressive proliferation, invasiveness, diffuse infiltration and often high resistance to anticancer drugs [1,2]. For this reason, the prognosis for patient survival is poor. Researchers have made great efforts to develop therapeutic modalities that eliminate the ability of cells to resist treatments and induce cell death [3].

Photodynamic therapy (PDT) is a modern trend of adjuvant therapy in which a photosensitive molecule—a photosensitizer (PS), light at an appropriate wavelength absorbed by the PS, and oxygen are involved in the photoreactions that lead to photodestruction of the tumor [4]. In recent years, PDT has been used clinically to cure lung, head and neck, brain, prostate, colon, pancreatic, cervical, breast, and skin cancers [5]. The penetration depth of light is a major obstacle in PDT [6,7]. Light sources emitting in the red and near-infrared parts of the electromagnetic spectrum can be used to enable excitation of PSs relatively deep in tissues [6]. In addition, interstitial optical fiber-based cylindrical light distributors are also sometimes used to deliver light into bulky lesions. Therefore, surgical intervention in combination with PDT is a promising approach for the treatment of glioblastomas [8].

Hypericin is an interesting PS, which can be used for both PDT and photodiagnosis of cancer [9]. Hypericin is a naphthodianthrone characterized by a high hydrophobicity, the formation of non-fluorescent aggregates in aqueous solutions and a fluorescence emission of its monomers around 600 nm [10,11]. Hypericin dissolves well in the lipidic environment found in cell membranes [12]. Photodamages are induced in cells by singlet oxygen produced during the photoreaction taking place between hypericin molecules excited in their triplet and molecular oxygen [13,14,15].

The molecular mechanisms of PDT involve, in particular, the triggering of signaling pathways that lead to apoptosis of cancer cells. We have previously reported that the protein kinase C (PKC) signaling pathway is involved in the apoptotic response of glioblastoma cells to hypericin-mediated PDT [16,17,18]. We also observed the activation of PKCα and phosphorylation of Bcl2 in U87 MG glioblastoma cells after hypericin-mediated PDT [17,19]. The subcellular localizations of hypericin have been identified: the Golgi apparatus, lysosomes, plasma membrane and, in a few reports, in mitochondria [16,20,21,22,23,24]. The influence of hypericin on Bax and Bak, members of the Bcl2 protein family, suggests that hypericin plays an important role in the regulation of mitochondrial functions [25,26].

It has been reported that photobiomodulation (PBM) with light at 808 nm has beneficial effects on damaged mitochondria in cells [27,28,29,30,31]. In recent years, PBM has become an increasingly attractive modality to modulate reactive oxygen species in cells [32]. An approach to reduce inflammatory and oxidative stress markers by PBM at 810 nm has been demonstrated in hair cells [33]. The ease of application of light in different tissues gave rise to the idea of combining PBM and PDT. PBM at 660 nm was used to treat oral mucosa in combination with curcumin-mediated PDT at 468 nm [34]. For example, antimicrobial PDT with methylene blue and PBM has been used to improve and accelerate the healing process in the treatment of palatal ulcers [35]. The combination of PBM and PDT has even been proposed as an innovative approach for COVID-19 treatments [36].

While previous studies using curcumin and methylene blue-mediated PDT focused on antimicrobial treatment and PBM aimed at healing damaged tissues, the present work aims to demonstrate the efficacy of PBM at 808 nm and hypericin-mediated PDT in treating U87 MG glioblastoma cells. In our approach, hypericin forms non-fluorescent aggregates in the cytoplasm of cells that cannot be used for PDT. The use of PBM prior to hypericin application results in the formation of vesicles associated with the plasma membrane (of lipidic origin), which help to transport and dissolve hypericin intracellularly so that it is in a biologically active/fluorescent form for PDT. This further increases the production of lactate dehydrogenase and the efficacy of PDT. Various methods for detecting the metabolic activity of cancer cells and visualizing morphological changes by confocal fluorescence microscopy were used to detect the differences between cells treated with hypericin-PDT and PBM-hypericin-PDT. The interplay of autophagy and apoptosis, the two main modes of cell death, was studied by flow cytometry and Western blot of apoptotic and autophagic markers in U87 MG cells.

## 2. Materials and Methods

### 2.1. Cell Culture and Therapeutical Protocols

U87 MG human glioblastoma cells (cell culture was obtained from Cells Lines Services, Eppelheim, Germany) were grown in DMEM (Dulbecco’s modified Eagle medium, high glucose, GlutaMAX^TM^, with pyruvate, Gibco-Invitrogen, Life Technologies Ltd., Paisley, UK) supplemented with 10% FBS (fetal bovine serum, Gibco-Invitrogen, Life Technologies Ltd., Paisley, UK) and 1% (*w*/*w*) penicillin/streptomycin (Gibco-Invitrogen, Life Technologies Ltd., Paisley, UK) to 80% confluency, in the dark, under humidified atmosphere, 5% CO_2_ and 37 °C.

**Figure 1:**
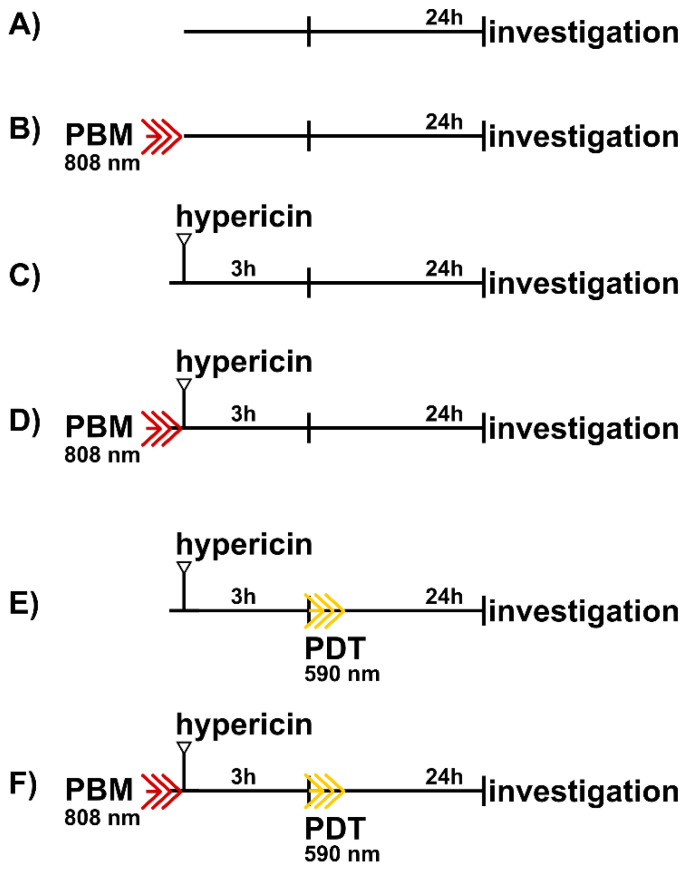
Schemes of therapeutical protocols: (**A**) control, (**B**) PBM, (**C**) hypericin in dark, (**D**) PBM-hypericin, (**E**) hypericin-PDT, and (**F**) PBM-hypericin-PDT.

Six therapeutical protocols were applied (see Figure 1). All protocols were stopped at the same time interval. PBM (808 nm diode laser (MDL-III-808/1~2500 mW, Changchun New Industries Optoelectronics Tech. Co. Ltd., Changchun, China), 2 min, 1.8 J/cm^2^, 15 mW/cm^2^) was applied shortly before hypericin administration into the cell culture medium. Hypericin at concentrations of 200 and 500 nM was administered for 3 h before PDT (590 nm light emitting diodes (homemade system), 2 min, 2 J/cm^2^, 16.7 mW/cm^2^). Cell responses were examined 5 and 24 h after PDT. Mitochondrial stress and damages to the Golgi apparatus were induced with 100 nM and 10 µM rotenone (Sigma-Aldrich, Darmstadt, Germany) for 24 h. Phorbol 12-myristate 13-acetate (PMA, Sigma-Aldrich) at a concentration of 1 µM was applied to stimulate protein kinase C.

### 2.2. Confocal Fluorescence Microscopy

U87 MG human glioblastoma cells were grown in confocal petri dishes embedded with cover slide (SPL, Gyeonggi-do, Korea). Mitochondria were labeled with 5 µM Rhodamine 123 (Rh123, Sigma-Aldrich, Darmstadt, Germany) for 15 min (excitation at 488 nm and emission in the spectral range 490–560 nm). Cell nuclei were labeled with 10 µg/mL Hoechst 33258 (ThermoFisher Scientific, Waltham, MA, USA) for 30 min (excitation at 405 nm and emission in the spectral range 450 ± 40 nm). Lysosomes were labeled with 400 nM LysoTracker Blue (ThermoFisher Scientific) (excitation at 405 nm and emission in the spectral range 450 ± 40 nm). NucView^®^ 488 caspase-3 substrate (Biotium, Fremont, CA, USA) was used to detect caspase level in cells according to the supplier protocol (excitation at 488 nm and emission in the spectral range 490–560 nm). Plasma membranes were stained with Cell Mask Orange (ThermoFisher Scientific) according to the supplier protocol (excitation at 488 nm and emission in the spectral range >560 nm). Hypericin was detected in the spectral range >590 nm after excitation at 555 nm. The fluorescence intensity profile was analyzed using fluorescence images where the intensity was normalized to the hypericin fluorescence in the dark without PBM. Images with increased fluorescence intensity (HI) and normalized to the brightest signal (pixel) in the image were used to identify hypericin localization in cells.

Giantin and Protein Interacting with C Kinase—1 (PICK1) were visualized in cells with immunostaining. Cells were fixed with ice-cold (−20 °C) acetone (Centralchem, Bratislava, Slovakia) for 5 min at −20 °C and washed in ice-cold phosphate-buffered saline (PBS, Sigma-Aldrich, Darmstadt, Germany). Cells were blocked in 5% bovine serum albumin (BSA, Sigma-Aldrich, Darmstadt, Germany) in PBS at room temperature (25 °C) for 1 h. The primary antibodies were dissolved in 5% BSA: anti-Giantin (1:300, ab80864, Abcam, Cambridge, UK) and PICK1 (1:300, Cell Signaling Technology, MA, USA) and incubated with the cells for 1 h at room temperature. After incubation, cells were washed with ice-cold PBS. The secondary antibody conjugated with AlexaFluor 488 (1:1000, ab150077, Abcam, Cambridge, UK) for Giantin and AlexaFluor 405 (1:1000, ab175652, Abcam, Cambridge, UK) for PICK1 were diluted in 1% BSA and applied to the cells for 1 h at room temperature. Fluorescence was detected in the following spectral domains: AlexaFluor 488 (excitation at 488 nm and emission in the spectral range 490–530 nm) and AlexaFluor 405 (excitation at 405 nm and emission in the spectral range 450 ± 40 nm).

Fluorescence images were acquired using a confocal fluorescence microscope system (LSM 700, Zeiss, Oberkochen, Germany), a 40× water immersion objective (NA 1.2, Zeiss), and a CCD camera (AxioCam HRm, Zeiss). Fluorescence images were analyzed using Zen 2011 software (Zeiss).

### 2.3. Cell Metabolism Assay and Lactate Dehydrogenase Assay

U87 MG human glioblastoma cells were seeded in 24-well plates. Cells were treated according to the protocols presented in Figure 1. After the treatments (24 h), 10 µL aliquots from the cell culture media were subjected to lactate dehydrogenase assay (LDH, Abcam) according to the supplier protocol. The absorbance of the LDH color assay was measured at 490 nm.

Detection of cellular metabolism was performed according to the supplier’s protocol. In this assay, 3-(4,5-dimethylthiazol-2-yl)-2,5-diphenyltetrazolium bromide (MTT, Sigma-Aldrich, St. Louis, MO, USA) is converted to purple formazan, which is then dissolved in dimethyl sulfoxide (DMSO, Sigma-Aldrich) and detected using 96-well plate reader (GloMax TM-Multi1Detection system with Instinct Software, Madison, WI, USA) at 560 nm. The mean values from 8 measurements were plotted in histograms. The error bars represent the standard deviations. The levels of significant differences were calculated using one way ANOVA-test: a (dark), b (PBM), c (PDT), d (PBM + PDT) < 0.05, * *p* < 0.05, ** *p* < 0.01, *** *p* < 0.001.

### 2.4. Flow Cytometric Assay

Human U87 MG glioblastoma cells were treated according to the protocols in Figure 1. Five hours after PDT, cells were collected by trypsin/EDTA (ThermoFisher Scientific) and centrifuged at 600 rpm. Cell pellets were resuspended in Annexin V binding buffer (Mitenyi Biotec B.V. & Co. KG, Bergisch Gladbach, Germany), into which AnnexinV/FITC (Mitenyi Biotec B.V. & Co. KG, Bergisch Gladbach, Germany) or NucView^®^ 488 caspase-3 substrate was added. Propidium iodide (PI, Mitenyi Biotec B.V. & Co. KG, Bergisch Gladbach, Germany) was added to the cell suspension just before detection by flow cytometer (MACSQuant^®^ Analyzer, Miltenyi, Bergisch Gladbach, Germany) in channels B1 and B3. Hypericin fluorescence was detected in B2 channel.

### 2.5. Western Blot Assay

Cells at a density of 10^6^ were lysed and homogenized in radioimmunoprecipitation buffer (RIPA) (150 mM sodium chloride, 1% Triton X-100, 0.5% sodium deoxycholate, 0.1% sodium dodecyl sulfate, 50 mM Tris, pH 8; all chemicals were purchased from Sigma-Aldrich, Darmstadt, Germany) with the inhibitor cocktail (2 × 1:100, Halt™ Protease and Phosphatase Inhibitor Cocktail, ThermoFisher Scientific, Waltham, MA, USA). Whole lysates (120 μg total protein amount) were diluted to 60 μg of final protein amount in 2× Laemmli buffer (Sigma-Aldrich, Darmstadt, Germany), loaded onto 7% or 15% polyacrylamide gels and subjected to electrophoresis. Proteins were transferred to a nitrocellulose membrane (0.22 μm; AppliChem, Darmstadt, Germany). Immunodetection was performed using the Western Breeze Chromogenic Kit (ThermoFisher Scientific, Waltham, MA, USA). Proteins in the membrane were blocked with 5% BSA for 1 h at room temperature, washed, and then incubated overnight at 4 °C with primary antibodies: anti-LC3B (1:3000, ab221794, Abcam, Cambridge, UK), anti-PKCα (1:3000, ab4124, Abcam, Cambridge, UK), anti-STAT3 (1:1000, ab109085, Abcam, Cambridge, UK) and anti-GAPDH (1:1000, ab181602, Abcam, Cambridge, UK) were determined as housing protein. For protein visualization, secondary antibodies from the Western Breeze Chromogenic Kit were used to detect the primary rabbit antibodies. Analysis of the optical densities (O.D.) of the proteins in the membrane was performed using ImageJ software [37]. The normalized O.D. values presented in the histograms are the means of 3 measurements. The error bars represent the standard deviations.

## 3. Results

### 3.1. PBM Decreases Cell Metabolic Activity and Increases LDH Activity after Hypericin-PDT of U87 MG Cells

**Figure 2:**
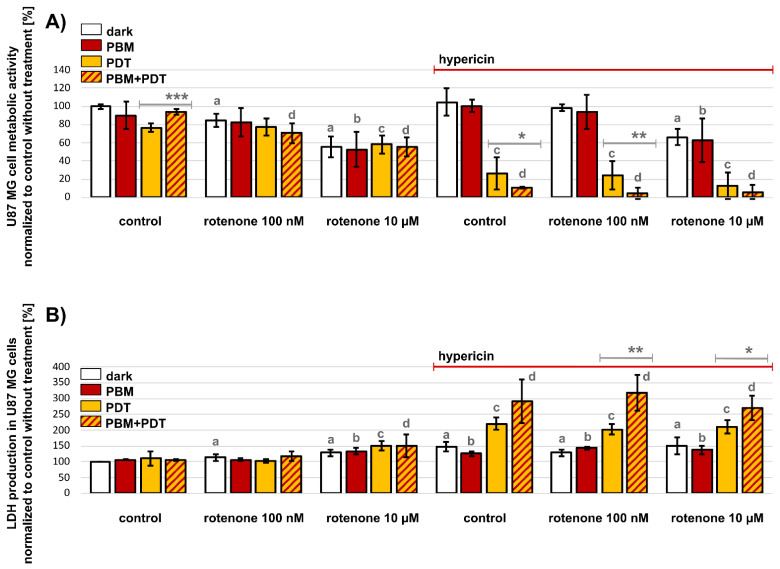
(**A**) Metabolic activity of U87 MG cells and (**B**) LDH activity in the dark (white columns), after PBM (red columns), hypericin-PDT (orange columns) and PBM-hypericin-PDT (orange columns with red patterns). Hypericin concentration was 500 nM (administered to cells for 3 h). Cell damage was also induced by 100 nM and 10 µM rotenone for 24 h. Significance level (against controls without hypericin treatment) was estimated using the one-way ANOVA-test: a (dark), b (PBM), c (PDT), d (PBM + PDT) < 0.05, * *p* < 0.05, ** *p* < 0.01, *** *p* < 0.001.

The metabolic activity of cells was detected in U87 MG glioblastoma cells after different treatment conditions. Two concentrations of rotenone were chosen to intoxicate the cells. It was found that the lower concentration of 100 nM rotenone inhibited complex I of the mitochondrial respiratory chain. At this concentration, the metabolic activity of the cells was not significantly affected compared to untreated controls (Figure 2A), although mitochondrial morphology was expected to undergo a fission. In contrast, 10 µM rotenone significantly decreased the metabolic activity of U87 MG cells (*** *p*). This concentration of rotenone leads not only to mitochondrial fission, but also to fragmentation of the Golgi apparatus, as later shown by confocal fluorescence microscopy. Neither PDT nor PBM affected the metabolic activity of the cells damaged by rotenone. Administration of 500 nM hypericin for 3 h in the dark and after PBM (see protocols in Figure 1) resulted in no significant differences compared with the untreated control. A significant decrease in the metabolic activity of U87 MG cells was only observed with the combined treatment of 10 µM rotenone for 24 h (Figure 2A). However, PDT of cells treated with hypericin (hypericin-PDT) resulted in quite dramatic changes. Indeed, the metabolic activity of treated cells decreased to below 50% compared to the activity of untreated control cells maintained in the dark. This effect was also observed with the combined treatment with both rotenone concentrations. In addition, PBM of U87 MG cells performed before hypericin administration (PBM-hypericin-PDT) increased the efficacy of PDT. The increased phototoxicity of PBM-hypericin-PDT was confirmed by the LDH assay, in which LDH production increased by up to 300% of untreated control cells (Figure 2B). A marked increase in LDH production was also observed in cells after hypericin-PDT. However, this effect was weaker (<250%) than that observed after PBM-hypericin-PDT. The presence of rotenone had no significant effect on hypericin-PDT and PBM-hypericin-PDT (Figure 2B).

### 3.2. PBM Decreases Hypericin Fluorescence in U87 MG Cells

**Figure 3:**
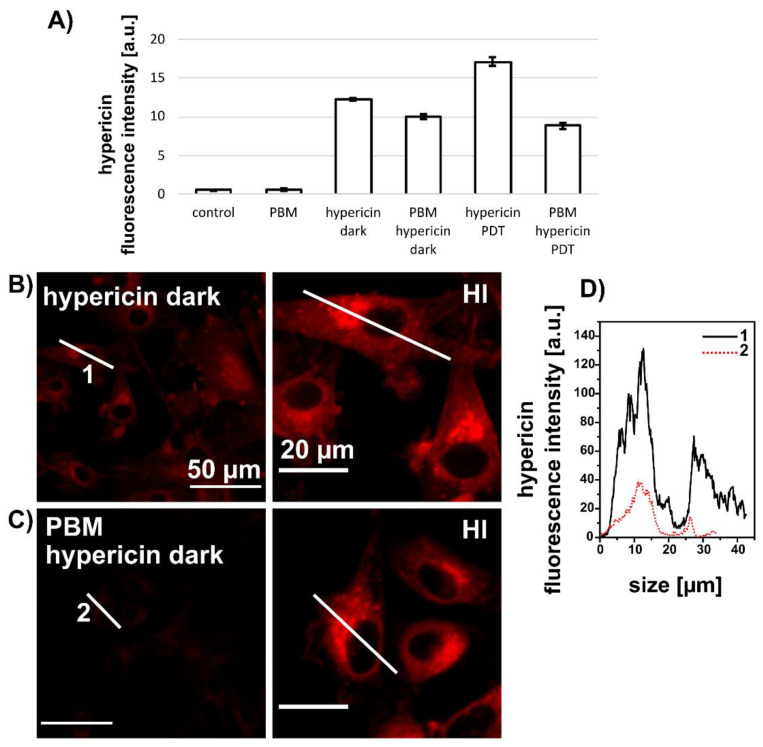
(**A**) Fluorescence intensity of 200 nM hypericin detected by flow cytometry in U87 MG cells under different conditions. Detection was performed 5 h after PDT. (**B**) Fluorescence distribution of 200 nM hypericin (3 h) in cells without and (**C**) with PBM. HI indicates high intensity correction to allow localization of hypericin. Scale bar—50 µm. White lines were drawn along selected cells to show the distribution of the hypericin fluorescence intensity in the cell along these lines. (**D**) Representative fluorescence distribution along lines 1 and 2 shown in (**B**,**C**), respectively.

The efficacy of PDT can be increased by increasing the concentration of hypericin. It can be assumed, in a first approximation, that the biological activity of hypericin is proportional to its fluorescent form in cells. Therefore, we studied the subcellular distribution of hypericin and its fluorescence in U87 MG cells. The hypericin concentration was reduced to 200 nM to decrease the phototoxic effect during the fluorescence measurements. The fluorescence intensity of 200 nM hypericin detected by flow cytometry in U87 MG cells 5 h after PDT is shown in Figure 3A. The fluorescence intensity of hypericin increased in cells after PDT (hypericin-PDT). In contrast, hypericin fluorescence decreased in cells treated with PBM-hypericin and PBM-hypericin-PDT. This observation was confirmed by confocal fluorescence microscopy (Figure 3B–D). The fluorescence of hypericin administered for 3 h in the dark (Figure 3B) was more intense than that of PBM-hypericin (Figure 3C). This difference is clearly seen in the hypericin fluorescence intensity profiles of selected cells (labeled 1 and 2 in Figure 3D). Hypericin fluorescence intensity correction (images labeled HI) and normalization to the brightest signal (pixel) in the image were used to identify the subcellular distribution of hypericin. In both treatments, a homogeneous localization of hypericin with bright intensity can be observed in the perinuclear region associated with the Golgi apparatus.

### 3.3. Lysosomes Degradation, Mitochondria Destabilization and Fragmentation of Golgi Apparatus Are Induced by Hypericin-PDT and PBM-Hypericin-PDT

**Figure 4:**
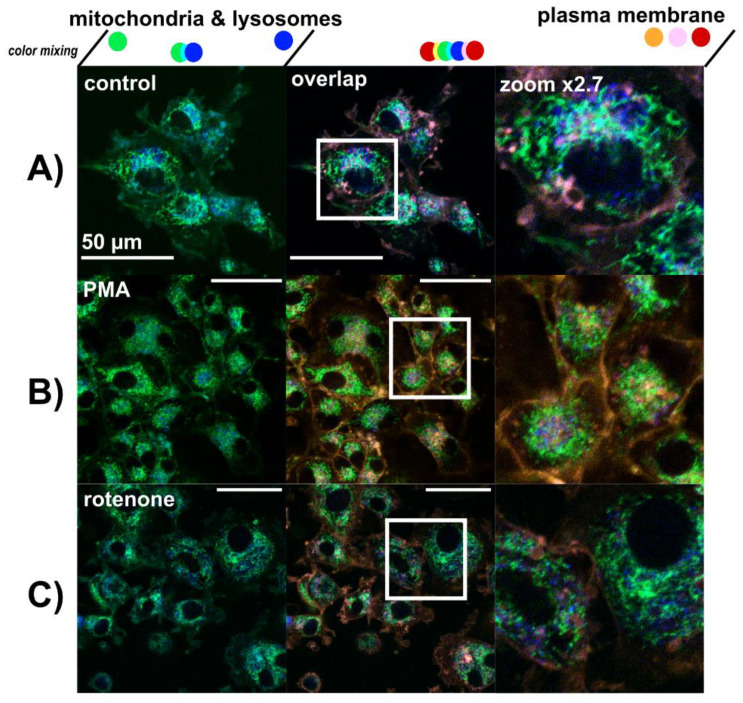
Confocal fluorescence images of rhodamine 123—mitochondria (green), LysoTracker Blue—lysosomes (blue), and Cell Mask Orange—plasma membrane (orange and red) in (**A**) U87 MG cells without treatment, (**B**) treated with 1 µM PMA, and (**C**) 10 µM rotenone for 5 h. The overlapping images were magnified to better see the organelles detected. Scale bar—50 µm.

The organelles of main interest in the present study were mitochondria, lysosomes, Golgi apparatus and plasma membrane. These organelles were stained with specific fluorescent probes as shown in Figure 4.

Two treatments were chosen: 1 µM PMA and 10 µM rotenone administered over 5 h. Under these conditions, mitochondrial and lysosomal oriented oxidative stress can be induced, as can be seen by the morphology of mitochondria, which is different from the control (Figure 4A). Increased numbers of lysosomes were also observed (Figure 4B–C). While mitochondria and lysosomes were stressed and centralized around the nucleus after PMA treatment (see zoom in Figure 4B), rotenone stimulated a scattered distribution of lysosomes and mitochondria in the cytoplasm (see zoom in Figure 4C).

**Figure 5:**
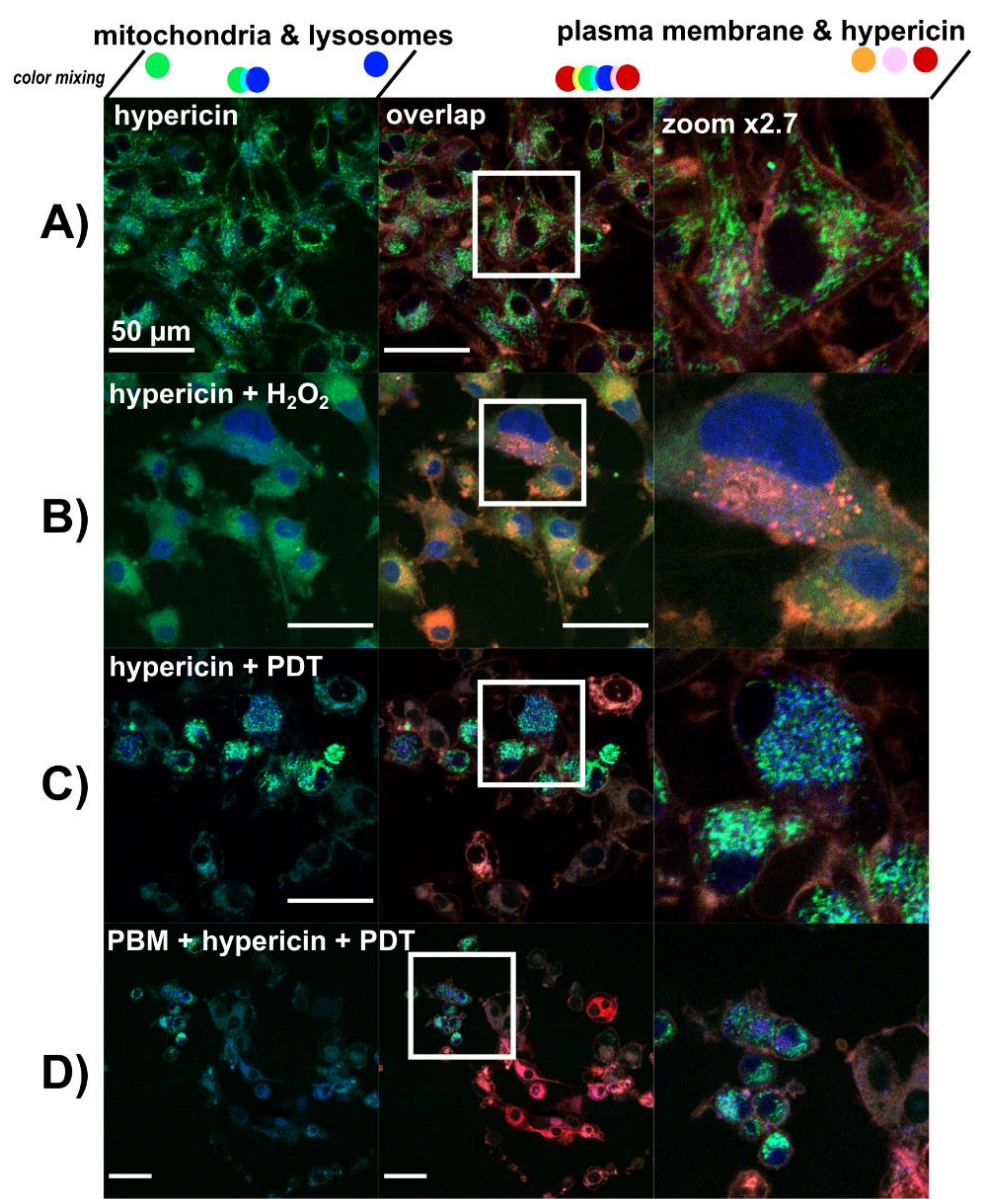
Confocal fluorescence images of rhodamine 123—mitochondria (green), LysoTracker Blue—lysosomes (blue), hypericin (orange and red) and Cell Mask Orange—plasma membrane (orange and red) in (**A**) U87 MG cells treated with 500 nM hypericin for 3 h, (**B**) hypericin in A + 1 mM H_2_O_2_ for 10 min, (**C**) hypericin-PDT and (**D**) PBM-hypericin-PDT detected 5 h after PDT. Overlapping images were magnified to better identify the organelles detected. Scale bar—50 µm.

The morphology and localization of selected organelles observed in U87 MG cells after hypericin treatment in the dark resembled the untreated control (Figure 5A). To confirm the changes induced by massive nonphysiological oxidative stress, hydrogen peroxide was administered to these cells (Figure 5B). Destabilization and degradation of lysosomes resulted in translocation of LysoTracker Blue (lysosomal probe—blue color) into the nuclei of U87 MG cells. The mitochondrial membrane potential was dissipated by hydrogen peroxide and the mitochondrial probe (rhodamine 123—green color) diffused into the cytoplasm.

Hypericin-PDT (Figure 5C) and PBM-hypericin-PDT (Figure 5D) triggered dramatic changes in the cells. Mitochondrial fission, an increase in the number of lysosomes, mitochondria membrane potential depletion, and lysosome destabilization were observed in the cells. When mitochondria and lysosomes could be observed, a perinuclear localization of these organelles was noted. If we subdivide the cells according to the stress induced by the previous treatments with PMA, rotenone and hydrogen peroxide, all three types of cell effects could be found after hypericin-PDT and PBM-hypericin-PDT. It should be noted that the red fluorescence consists of hypericin and Cell Mask Orange signals bleeding into the same detection channel.

**Figure 6:**
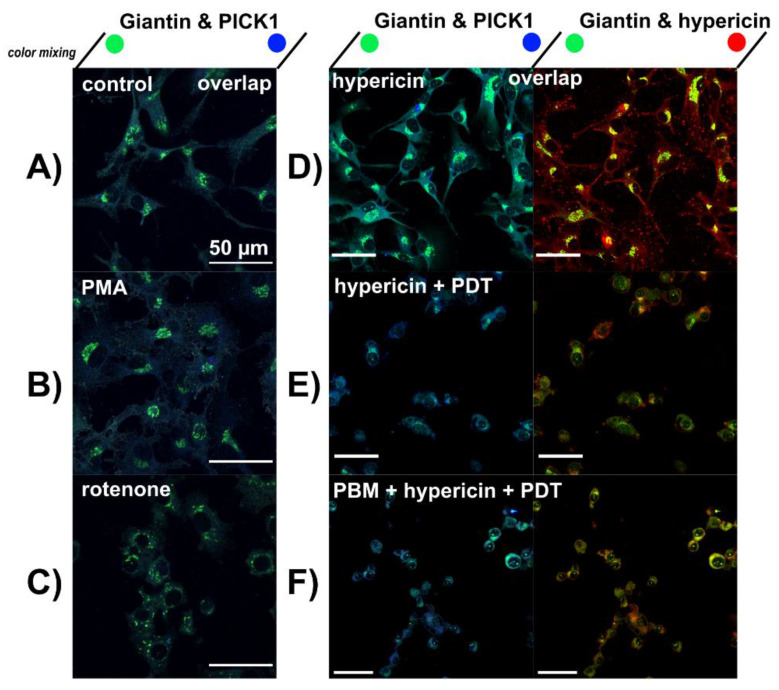
Confocal fluorescence images of Giantin (green), PICK1 (blue) and hypericin (red) in (**A**) U87 MG cells without treatment, (**B**) treated with 1 µM PMA and (**C**) 10 µM rotenone for 5 h. (**D**) cells treated with 500 nM hypericin for 3 h, (**E**) hypericin-PDT and (**F**) PBM-hypericin-PDT detected 5 h after PDT. Scale bar—50 µm.

The localization of hypericin was observed in the Golgi apparatus. For this reason, we immunostained Giantin localized in the Golgi apparatus of U87 MG cells. For better identification, a skeleton of U87 MG cells was detected with an antibody against PICK1. Beautiful cisternae of the Golgi apparatus localized in the perinuclear region can be detected in untreated control (Figure 6A), PMA (Figure 6B) and hypericin (Figure 6D) treated cells. Hypericin can be detected by its red fluorescence. Fragmentation of the Golgi apparatus and scattered localization of cisternae in the cytoplasm were observed in cells treated with rotenone (Figure 6C). Destabilization and fragmentation of the Golgi apparatus was also observed after hypericin-PDT and PBM-hypericin-PDT cells.

### 3.4. PBM Increases Autophagy in U87 MG Cells after Hypericin-PDT

**Figure 7:**
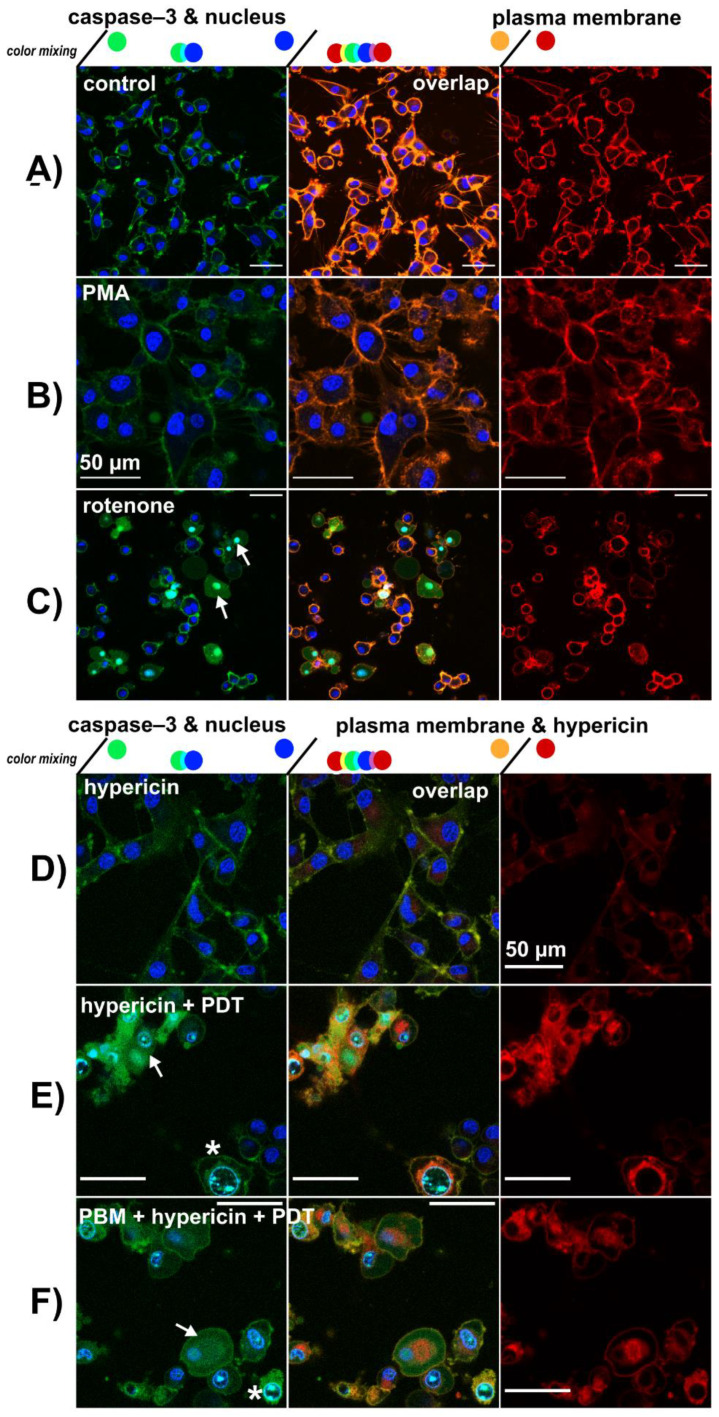
Confocal fluorescence images of NucView^®^ 488 caspase-3 substrate (green), Hoechst—nucleus (blue), hypericin (red) and Cell Mask Orange—plasma membrane (orange and red) in (**A**) U87 MG cells without treatment, (**B**) treated with 1 µM PMA and (**C**) 10 µM rotenone for 5 h. (**D**) U87 MG cells treated with 500 nM hypericin for 3 h, (**E**) hypericin-PDT and (**F**) PBM-hypericin-PDT detected 5 h after PDT. Scale bar—50 µm. White asterisks indicate cells with typical apoptotic features stained with Hoechst. White arrows indicate localization of NucView^®^ 488 caspase-3 substrate in the cytoplasm and nucleus.

While the cells in the untreated control and hypericin treatment in the dark have the typical shape of U87 MG glioblastoma cells, those irradiated (PDT) have a round shape. Such a shape is typical of cells undergoing apoptosis and death. Subcellular localization of the NucView^®^ 488 caspase-3 substrate was imaged in U87 MG cells treated as in the previous Section 3.3. These cells were stained with Cell Mask Orange and Hoechst to reveal the plasma membrane and nucleus. This combination led to the visualization of green fluorescence in the plasma membrane originating from Cell Mask Orange (see Figure 7A,B). On the other hand, cytosolic localization of green fluorescence belongs to the NucView^®^ 488 caspase-3 substrate as observed in cells after rotenone treatment (Figure 7C). Typical apoptotic nuclei stained with Hoechst are indicated by white arrows in Figure 7C.

Hypericin-PDT and PBM-hypericin-PDT resulted in cytosolic localization of the NucView^®^ 488 caspase-3 substrate and condensation of chromatin visualized with Hoechst (Figure 7E,F). This was not observed in cells treated with hypericin in the dark (Figure 7D).

**Figure 8:**
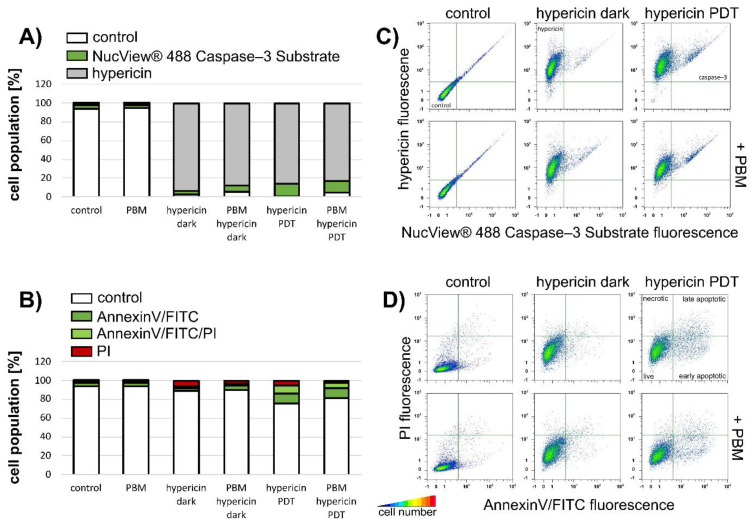
Flow cytometric analysis of (**A**) caspase-3 production (stained with NucView^®^ 488 caspase-3 substrate (green histograms)) and (**B**) apoptosis/necrosis (stained with AnnexinV/FITC (green histograms) and PI (red histograms)) in U87 MG cells without treatment and treated with PBM, 200 nM hypericin in the dark, after PBM and PDT, as shown in the histograms. Detection was performed 5 h after PDT. White histograms show cells without staining and gray histograms show cells labeled with hypericin. (**C**) Correlation plots of the fluorescence intensities of hypericin and NucView^®^ 488 caspase-3 substrate in the detected cells. (**D**) Correlation plots for the intensity of fluorescence of PI and AnnexinV/FITC in the detected cells. Cell number is color-coded (blue—minima, red—maxima).

The increase of NucView^®^ 488 caspase-3 substrate in U87 MG cells after hypericin-PDT and PBM-hypericin-PDT was confirmed by flow cytometry (green histograms in Figure 8A,C). Apoptotic cell populations with AnnexinV/FITC and PI staining were observed after these treatments (Figure 8B,D).

It should be noted that flow cytometry, in contrast to confocal fluorescence microscopy, was performed on cells treated with 200 nM hypericin to reduce the number of completely damaged/fragmented cells after PDT.

**Figure 9:**
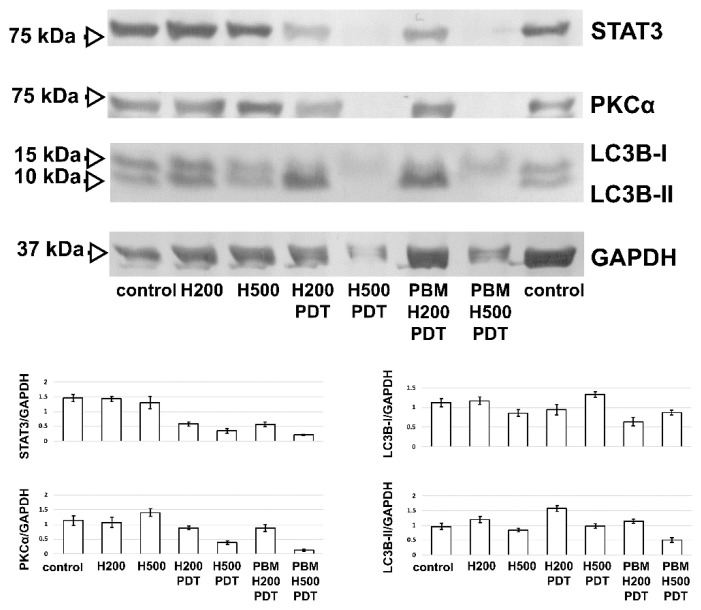
Western blot analysis of PKCα, STAT3 and LC3B protein levels in U87 MG cells without treatment and treated with PBM, 200 nM (H200) and 500 nM (H500) hypericin in the dark, after PBM and PDT, as shown in the images and histograms. GAPDH was used as the housing protein. The histograms show the optical densities of the detected bands.

Since STAT3, LC3B and PKCα are involved in cancer cell migration, autophagy and apoptosis, Western blot analysis of their protein levels in cells was performed. The results are shown in Figure 9. The protein levels of STAT3 and PKCα decreased in the cells treated with 200 nM hypericin after PDT and PBM-hypericin-PDT. The bands decreased in the cells treated with 500 nM hypericin after PDT and PBM-hypericin-PDT.

LC3B is divided into two bands: LC3B-I and LC3B-II. Both LC3B bands were detected in the untreated control and hypericin-treated cells. However, LC3B-II was increased in the cells treated with 200 nM hypericin-PDT and PBM-hypericin-PDT (Figure 9). In contrast, LC3B-I was present in the cells treated with 500 nM hypericin-PDT and PBM-hypericin-PDT.

## 4. Discussion

The anti-glioblastoma activity of hypericin has been demonstrated without light application [38]. It has been suggested that hypericin may cause epigenetic changes, modulation of neuroglial tumor cell differentiation, modifications of the cytoarchitecture, and cell cycle alterations. Moreover, hypericin is known to inhibit the activity of PKC, which is involved in proliferation and cell death [39,40]. Therefore, the increase of its intracellular concentration by PBM could contribute to the enhancement of its biological activity in the dark and even improve its proapoptotic function in cancer cells after PDT.

Five proposed treatments were evaluated in this study. Only PBM was applied to improve glioblastoma cell status without administration of a photosensitizer (hypericin). The combination of PBM with hypericin without further illumination (no PDT) was compared with the biological activity of hypericin in the dark. No significant differences in the metabolic activities (detected with MTT) of these cells were observed, but some significant increase in LDH production was observed. This may be attributed to an increased oxygen consumption during PBM, which may temporarily switch the cell to anaerobic glycolysis. This effect was also observed in cells treated with hypericin-PDT, where LDH increased tremendously due to oxygen consumption. Consequently, PBM-hypericin-PDT resulted in the best treatment effect, i.e., proliferation was inhibited and LDH increased significantly. This effect was even better observed in the cells to which rotenone, an inhibitor of mitochondrial respiration, was applied. The destruction and alteration of subcellular organelles by hypericin-PDT and PBM-hypericin-PDT provided the impetus for autophagy and apoptosis signaling.

PDT has been shown to modulate the effect of hypericin on PKC isoform activity in glioblastoma cells [16,17]. This signaling molecule was also investigated in the present study. It was found that PKCα isoform, which is considered as an anti-apoptotic factor, was strongly affected by PDT. The protein level of PKCα decreased in glioblastoma cells after PDT depending on the concentration of hypericin. While PBM did not affect 200 nM hypericin-PDT, a higher concentration of 500 nM hypericin-PDT resulted in a greater reduction of PKCα in glioblastoma cells pretreated with PBM (PBM-hypericin-PDT). It should be noted that the drastic decrease in PKCα protein level is related to the translocation of PKCα from the cytosol to the plasma membrane, where it plays an important role in the apoptotic response of cancer cells. PBM was also shown to increase PKCα protein levels in untreated U87 MG cells and maintain PKCα localization in the perinuclear region.

The protein Signal Transducer and Activator of Transcription (STAT)-3, which is involved in the protection of cells from apoptosis, was also identified as a protein regulated by PKC [41,42]. In the present study, STAT3 protein level in U87 MG cells decreased similarly to PKCα after hypericin-PDT and PBM-hypericin-PDT. Downregulation and inhibition of STAT3 after PDT leading to apoptosis was observed in cancer cells treated with 5-aminolevulinic acid, 2-[1-hexyloxyethyl]-2devinyl pyropheophorbide-a and benzoporphyrin derivative [43,44,45]. This inhibition has been associated with inhibition of proliferation, infiltration of cancer cells, and induction of apoptosis in solid tumors.

Several studies have reported that hypericin-PDT induces apoptosis through the activation of caspase-3 and the release of cytochrome c with the recruitment of BH3-interacting domain death agonists (Bid, Bax, Bac, Bcl2) [25,46,47]. In our study, both hypericin-PDT and PBM-hypericin-PDT led to an increase in caspase-3 and apoptosis. However, our fluorescence microscopy results showed that not only mitochondria but also lysosomes and Golgi apparatus were strongly affected by hypericin-PDT.

The process of autophagy is often triggered in cancer, inflammation and neurodegeneration to maintain homeostasis in the cell, especially during the degradation of lysosomes [48,49]. In this process, LC3 plays an important role. In particular, lipidated LC3-II is associated with the formation of double-membrane autophagosomes [50]. We have shown here that 200 nM hypericin-PDT increased LC3B-II and 500 nM hypericin-PDT increased LC3B-I protein levels in U87 MG cells. Application of PBM prior to PDT reduced the effects observed in cells without PBM. Therefore, our results suggest that PBM-hypericin-PDT promotes apoptosis before autophagy. However, the balance between autophagy and apoptosis may be modulated by hypericin concentration in addition to PBM. The reduction in hypericin fluorescence intensity observed by flow cytometry and fluorescence microscopy after PBM may be explained by PBM-induced detoxification. We hypothesize that PBM-induced autophagy may be related to the detoxification processes in the cell.

As mentioned in the introduction, PBM has already been used in combination with PDT, but the aim of PBM application was to heal the treated area [34,35]. Enhancement of endogenous protoporphyrin IX production and homogenization in U87 MG cells by PBM has recently been addressed as a promising protocol for PDT and photodiagnostics [51]. To the best of our knowledge, this is the first time that PBM has been used in combination with PDT to eliminate cancer cells with photoactivated hypericin.

In our study, we showed that glioblastoma cells strive to reduce the photodamages induced by PBM-hypericin-PDT. However, metabolic activity and LDH levels in cells were significantly and much more severely affected by PBM-hypericin-PDT than by hypericin-PDT. Since LDH is important for both anaerobic metabolism and glycolysis, the effect of PBM may be attributed to oxygen consumption and glycolysis. The treatment efficacy was also higher in cells with damaged mitochondria and a Golgi apparatus stimulated with rotenone. This suggests that the elimination of cells by apoptosis may be enhanced by PBM-PDT in the case of mitochondrial and Golgi apparatus dysfunction, which is expected under less oxygen-rich conditions.

In conclusion, the application of PBM in our conditions was such that hypericin was not excited by the PBM light, but the biological activity of hypericin after PDT was affected by PBM. While PBM alone stimulates the detoxification of glioblastoma cells and decreases the hypericin intensity, consequently, probably due to its intracellular concentration, faster than its natural release, application of PDT to cells pretreated by PBM results in a more potent destructive effect than hypericin-PDT alone. Therefore, we hypothesize that this combination of light treatments led to an improvement in hypericin-PDT efficacy and to the identification of this PBM-hypericin-PDT combination as a promising treatment for glioblastomas. This treatment strategy is probably also of interest for solid tumors with heterogeneous metabolism and oxygenation.

## Data Availability

Data are presented in this work.
